# Risk factors for poor outcome in childhood tuberculous meningitis

**DOI:** 10.1038/s41598-021-87082-5

**Published:** 2021-04-21

**Authors:** Mao-Shui Wang, Mei Zhao, Xin-Jie Liu

**Affiliations:** 1 Department of Pediatrics, Qilu Hospital, Cheeloo College of Medicine, Shandong University, 107#, Wenhuaxi Road, Jinan, 250012 Shandong China; 2 Department of Lab Medicine, Shandong Provincial Chest Hospital, Cheeloo College of Medicine, Shandong University, Jinan, Shandong China; 3 Department of Pediatrics, Shandong Maternal and Child Health Hospital, Jinan, Shandong China

**Keywords:** Paediatric research, Meningitis, Tuberculosis

## Abstract

Tuberculous meningitis (TBM) remains a serious disease for children and its risk factors of poor outcome remain unclear. Therefore, a retrospective study was conducted aiming to investigate the risk factors associated with poor outcome of childhood TBM. Between January 2006 and December 2019, consecutive children patients (≤ 15 years old) who had a diagnosis of TBM were included for the analysis. The demographic, clinical, laboratory, and radiographic data were collected from the electronic medical records retrospectively. Poor outcome was defined as death or transfer to a higher-level hospital. Patients were then divided into good and poor outcome groups. Subsequently, risk factors for poor outcome were estimated using univariate and multivariate logistic regression analysis. A total of 149 children with TBM was enrolled, twenty-two patients suffered poor outcome, including 16 transfers to a higher-level hospital and 6 deaths, and the remaining 127 patients were classified as good outcome group. Further multivariate analysis revealed that coma (age- and sex-adjusted OR = 6.425, 95% CI: 1.743, 23.676; *P* < 0.01) and cerebrospinal fluid (CSF) protein (> 1188.3 mg/L; age- and sex-adjusted OR = 4.680, 95% CI: 1.469, 14.902; *P* < 0.01) were associated with the poor outcome of childhood TBM. Childhood TBM remains to have a high mortality rate in China. High CSF protein and coma were identified as risk factors for poor outcome of childhood TBM. Hence, more attention is required to be paid to suspected patients with such characteristics, thus facilitating access to optimum treatment.

## Introduction

Currently, the situation of tuberculosis (TB) control in children has undergone many changes. First, the leading infectious agents causing death in children have shifted from rabies virus and *M.tuberculosis* to human immunodeficiency virus^1^. Second, during the past years, the annual notified incidence of childhood pulmonary TB was low (compared with that of adults) and decreased significantly^2^. However, TB caused by *M.tuberculosis* infection remains the most common bacterial infection in children and a serious public health threaten in China.

Tuberculous meningitis (TBM) is the most severe form of TB, leading to a high rate of mortality. In children, mortality rates of 15–29% were reported for TBM^3–5^. Recently, our findings demonstrated that the proportion of TBM among childhood TB in Shandong, China, is estimated at 9.4%, a remarkably high value required for effective intervention^6^. In addition, during the last decade, the trend in the proportion of TBM among childhood TB suggested a steady-state^6^. Therefore, a better management of childhood TBM is required to improve the current situation of TB patients, especially the survival rate.

It is well-known that delays in initiating therapy for TBM would increase mortality^7^. Unfortunately, the diagnosis of TBM in children is difficult. For example, routine microbiological examinations (such as smear and culture) show limited capacity in the diagnosis of the disease among children; polymerase chain reaction (PCR) has a moderate sensitivity in the diagnosis^8^, and a similar result was reported, when cerebrospinal fluid (CSF) Xpert was applied^9^; metagenomic next-generation sequencing is also tested in the diagnosis of TBM and presented with a moderate diagnostic performance^10^. The diagnosis remains a clinical challenge and this would contribute a significant delay in the initiation of anti-TB therapy, which may lead to a poor outcome. Hence, to characterize the risk factors associated with the poor outcome may improve the present situation in the management of childhood TBM.

The current study aims to investigate the risk factors for a poor outcome of TBM in children. There are few studies that address the factors associated with the outcome of TBM in this population. Our findings may be helpful in understanding the clinical characteristics of children patients with poor outcomes, and may improve the management of the disease.

## Materials and methods

The study was approved by the Ethics Committee of Shandong Provincial Chest Hospital, Jinan, China (NO. 2020XKYYEC-29) and performed in compliance with Helsinki declaration. Due to the retrospective nature of the study design and anonymous data collection, written informed consent was waived by the Ethics Committee of Shandong Provincial Chest Hospital.

Between January 2006 and December 2019, consecutive children patients (≤ 15 years old) who had a diagnosis of TBM were included for the analysis. TBM was defined if one of the following criteria (modified from the criteria established by Marais et al.^11^) was met: (1) definite: acid fast bacilli (AFB, +) on cerebrospinal fluid (CSF) microscopy, or CSF TB-PCR( +), or *M.tuberculosis* cultured from CSF. (2) conclusive: symptoms and signs of meningitis and CSF findings (such as total white cell count > 5 cells × 10^6^/L, protein > 0.45 g/L, glucose < 2.2 mmol/L, and CSF/serum glucose ratio < 0.5), *plus* at least one of the following (i) TB suggested by abnormal radiographic features (chest, or cerebral imaging), (ii) positive TB assays (such as AFB, PCR, and culture) using non-CSF samples. All patients were censored at death or discharge from our center.

The demographic, clinical, laboratory, and radiographic data were collected from the electronic medical records retrospectively. Poor outcome was defined as death or transfer to a higher-level hospital. Then, patients were divided into good and poor outcome groups and analyzed. Subsequently, risk factors for poor outcome were estimated in the study.

Statistical analysis were performed using SPSS version 16.0 (SPSS, Chicago, IL, USA). Continuous variables were expressed as mean ± standard deviation (SD) and categorical variables as count (percentages). Univariate and multivariate logistic regression analysis adjusted by age and sex were performed to determine risk factors for poor outcomes, and odds ratios (OR) and the corresponding 95% confidence interval (CI) were calculated^12^. Additionally, to make a better clinical understanding, continuous variables were transformed into categorical variables, based on cutoffs determined by the receiver operating characteristic curve (ROC) analysis. The goodness-of-fit was assessed using the Hosmer–Lemeshow test. A *P* value < 0.05 was considered significant.

## Results

### Patient characteristics

A total of 149 children was enrolled during the study period. Twenty-two patients suffered poor outcomes, including 16 transfers to a higher-level hospital and 6 deaths. Table 1 shows a comparison of clinical-pathological characteristics between patients with poor and good outcomes.

**Table 1:** Univariate analysis of the demographic data associated with poor outcome in childhood tuberculous meningitis.

N	Total (n)	Poor group (n)	Good group (n)	*P* value	OR (95% CI)
	149	22	127		
**Demographic characteristics**					
Age (years)	7.8 ± 5.3	6.9 ± 5.5	8.0 ± 5.2	0.357	
Sex (male)	82 (55.0%)	11 (50.0%)	71 (55.9%)	0.608	
**Vital signs**					
Temperature (°C)	37.3 ± 0.9	37.4 ± 0.9	37.3 ± 0.9	0.601	
Heart rate (beats/min)	99.2 ± 21.5	103.6 ± 29.7	98.5 ± 19.9	0.787	
Respiratory rate (breaths/min)	22.6 ± 3.0	23.2 ± 3.4	22.5 ± 2.9	0.818	
Systolic pressure (mmHg)	106.9 ± 16.1	114.4 ± 15.1	105.4 ± 15.9	0.031	1.025 (1.002, 1.048)
Diastolic pressure (mmHg)	68.9 ± 11.5	73.3 ± 12.8	68.0 ± 11.2	0.078	
**Medical history**					
Contact history of TB	32 (21.5%)	6 (27.3%)	26 (20.5%)	0.475	
Transferred from a teaching hospital	128 (85.9%)	18 (81.8%)	110 (86.6%)	0.552	
Frequency of hospitalization	2.1 ± 1.7	1.2 ± 0.7	2.2 ± 1.8	0.030	0.437 (0.207, 0.924)
Treatment delay (days)	40 ± 61	43 ± 43	40 ± 64	0.807	
Self-treatment	20 (13.4%)	6 (27.3%)	14 (11.0%)	0.046	3.027 (1.017, 9.005)
Out-patient history	87 (58.4%)	13 (59.1%)	74 (58.3%)	0.942	
Inpatient history	143 (96.0%)	21 (95.5%)	122 (96.1%)	0.893	
Antibiotics therapy	114 (76.5%)	19 (86.4%)	95 (74.8%)	0.247	
Anti-TB therapy	31 (20.8%)	3 (13.6%)	28 (22.0%)	0.375	
**Symptoms**					
Cough	30 (20.1%)	3 (13.6%)	27 (21.3%)	0.415	
Fever	132 (88.6%)	19 (86.4%)	113 (89.0%)	0.722	
Vomitting	72 (48.3%)	13 (59.1%)	59 (46.5%)	0.277	
Headache	77 (51.7%)	9 (40.9%)	68 (53.5%)	0.277	
Coma	22 (14.8%)	8 (36.4%)	14 (11.0%)	0.004	4.612(1.645, 12.932)
Drowsiness	14 (9.4%)	1 (4.5%)	13 (10.2%)	0.412	
Convulsion	25 (16.8%)	5 (22.7%)	20 (15.7%)	0.422	
Dizziness	5 (3.4%)	1 (4.5%)	4 (3.1%)	0.739	
Symptoms related to cranial nerve involvement*	16 (10.7%)	4 (18.2%)	12 (9.4%)	0.270	
**Cerebrospinal fluid analysis**					
White blood cell (10^6^/L)	156.2 ± 283.5	71.8 ± 109.4	170.9 ± 301.8	0.114	
Mononuclear cell (%)	76 ± 25	76 ± 24	76 ± 25	0.649	
Polyonuclear cell (%)	25 ± 25	25 ± 24	24 ± 25	0.947	
Lactate (mmol/L)	4.56 ± 1.99	5.28 ± 2.00	4.42 ± 1.97	0.235	
Total protein (g/L)	984 ± 758	1412 ± 1110	917 ± 670	0.019	1.001 (1.000, 1.001)
Aspartate aminotransferase (U/L)	15 ± 11	20 ± 14	14 ± 10	0.061	
Total bilirubin (mmol/L)	1.01 ± 2.57	1.21 ± 1.37	0.98 ± 2.71	0.739	
Adenosine deaminase (U/L)	5.5 ± 10.8	4.2 ± 4.4	5.7 ± 11.5	0.617	
Lactate dehydrogenase (U/L)	69 ± 103	107 ± 113	63 ± 101	0.135	
α-hydroxybutyrate dehydrogenase (U/L)	45 ± 55	72 ± 77	40 ± 50	0.047	1.007 (1.000, 1.014)
Total cholesterol (mmol/L)	0.15 ± 0.44	0.22 ± 0.46	0.14 ± 0.44	0.505	
Glucose (mmol/L)	2.01 ± 1.06	1.79 ± 0.78	2.05 ± 1.10	0.329	
IgA (mg/L)	18.38 ± 13.51	18.91 ± 17.26	18.16 ± 12.23	0.042	1.043 (1.001, 1.085)
IgG (mg/L)	80.63 ± 31.52	80.24 ± 35.38	80.79 ± 30.83	0.966	
IgM (mg/L)	4.28 ± 1.19	4.18 ± 1.29	4.32 ± 1.17	0.773	
Chloride (mmol/L)	113.3 ± 7.2	108.8 ± 5.5	114.0 ± 7.2	0.231	
Microbiological assays** (+)	30 (20.1%)	5 (22.7%)	25 (19.7%)	0.835	

*TB*, tuberculosis, *OR*, odds ratio, *CI* confidence interval. *Symptoms related to cranial nerve involvement include motor abnormalitie, speech difficulties, swallowing difficulties, and visual loss. **Microbiological assays include CSF AFB smear, CSF PCR, CSF Xpert, and CSF culture (“ + ” means at least 1 assay (+)).

In general, 22 patients were grouped as poor outcome group, and the remaining 127 patients as good outcome group. The mean age and weight were 7.8 ± 5.3 years and 24.2 ± 16.0 kg, respectively. Of them, eighty-two patients (55.0%) were male and 120 (80.5%) were from rural areas. Additionally, other sites involved with TB were reported as pulmonary (45.0%), miliary (15.4%), lymph node (4.0%), and pleural (4.0%). Among them, 30 patients (good outcome group, n = 25; poor outcome group, n = 5) have been confirmed with TBM: CSF culture (+ , n = 24); CSF AFB (+ , n = 2); TB PCR (+ , n = 13); CSF Xpert (+ , n = 2).

The mean times of hospitalization were 2.1 ± 1.7, and the mean treatment delay was reported at 40 ± 61 days. Among the enrolled patients, 32 patients (21.5%) had a TB contact history and 128 patients (85.9%) were transferred from a teaching hospital. Almost all patients (96.0%) had inpatient therapy, over half (58.4%) had outpatient therapy, and a significant proportion (13.4%) were reported having self-treatment. In addition, a majority (76.5%) of patients had experienced antibiotics therapy and a few (20.8%) were administrated with anti-TB therapy.

The vital signs were as follows: temperature, 37.3 ± 0.9 °C; heart rate, 99.2 ± 21.5 beats/min; respiratory rate, 22.6 ± 3.0 breaths/min; systolic pressure, 106.9 ± 16.1 mmHg; diastolic pressure, 68.9 ± 11.5 mmHg. The symptoms were as follows: fever (88.6%), headache (51.7%), vomiting (48.3%), cough (20.1%), convulsion (16.8%), coma (14.8%), drowsiness (9.4%), and dizziness (3.4%). In addition, a total of 16 patients (10.7%) were reported with symptoms related to cranial nerve involvement: motor abnormalities (n = 7), speech difficulty (n = 4), swallowing difficulty (n = 1), and visual loss (n = 4).

T-SPOT.TB were tested in 38 patients and 26 (68.4%) of them had a positive result. Other lab examinations, such as blood cell count, flow cytometry, and CSF analysis, were shown in Table 1 and Supplementary Table 1.

### Univariate and multivariate analysis

Table 1 shows the results of univariate analysis between poor and good outcome groups. It was found that poor outcome was associated with systolic pressure (OR = 1.025, 95% CI: 1.002, 1.048), frequency of hospitalization (OR = 0.437, 95% CI: 0.207, 0.924), self-treatment (OR = 3.027, 95% CI: 1.017, 9.005), CSF protein (OR = 1.001, 95% CI: 1.000, 1.001), CSF α-hydroxybutyrate dehydrogenase (α-HBDH, OR = 1.007, 95% CI:1.000, 1.014), and CSF IgA (OR = 1.043, 95% CI: 1.001, 1.085; all *P* < 0.05).

Further multivariate analysis (Hosmer–Lemeshow goodness-of-fit test: χ^2^ = 8.034, df = 8, *P* = 0.430) revealed that coma (age- and sex-adjusted OR = 6.425, 95% CI: 1.743, 23.676; *P* < 0.01) and CSF protein (> 1188.3 mg/L; age- and sex-adjusted OR = 4.680, 95% CI: 1.469, 14.902; *P* < 0.01) were associated with the poor outcome of childhood TBM (Table 2).

**Table 2:** Age- and sex-adjusted OR for risk factors associated with poor outcome in childhood tuberculous meningitis.

	Adjusted (age and sex) OR	*P* value
Coma	6.425 (1.743, 23.676)	0.005
CSF protein (> 1188.3 mg/L)	4.680 (1.469, 14.902)	0.009

*TBM* tuberculous meningitis, *OR* odds ratio, *CI* confidence interval.

## Discussion

To our knowledge, this study is the first assessing the risk factors associated with poor outcome in childhood TBM in China. Our findings were based on a moderate sample size and suggested that an increased level of CSF protein and coma were associated with the poor outcome in children with TBM. TBM treatment is more difficult and prone to failure than pulmonary TB. Our data may be useful to improve the current situation of childhood TBM.

Delays in treatment have limited influence on the outcome of the disease. As reported previously, delay in the treatment of TBM was associated with a poor outcome^13^. Besides, similar findings were also found by Verdon et al.^14^, and Sheu et al.^15^. However, these findings disagreed with our findings, our data demonstrated that no significant difference was observed in treatment delay between the poor and good outcome groups. Nevertheless, in the study, the treatment delay remains strongly suspected. Frequency of hospitalization was found to be associated with poor outcome in univariate analysis. On the one hand, the frequency of hospitalization may be associated with a prolonged period seeking an appropriate treatment. On the other hand, transfers between hospitals are more prone to support that these individuals have a more serious situation due to a delayed diagnosis or inappropriate treatment. Additionally, self-treatment also appeared to play a role in the treatment delay. However, this fact was not included in the final regression model.

Not surprisingly, coma is identified as a risk factor of poor outcome in our study. This may be explained that poor outcomes in TBM were largely confined to cases presenting in an advanced stage^16^. Similar observations were reported in several studies^13,17,18^. In our study, 22 (14.8%) of them had coma on arrival. In Romania, coma was noted in a similar proportion^19^. However, in low TB burden countries, this appears to be rare^20^. Although Glasgow coma scale has been introduced to evaluate the severity and predict the outcome of TBM^21^ and a low Glasgow coma scale (GCS) score on admission was associated with a poor prognosis, the accurate criteria (score cut-off) for this is not well characterized. For example, GCS (score: 3–8) is thought as an independent predictor of poor outcome in TBM patients^22^; the scale with ≤ 10 has a poor outcome^23^. In contrast, compared with Glasgow coma scale, coma as a symptom may be easy to use in the management of childhood TBM. Besides, if only death is defined as poor outcome, coma remains to be a risk factor for poor outcome of childhood TBM (data not shown).

Usually, a high level of CSF protein indicates disruption of the blood–brain barrier and the subarachnoid blockage of the CNS circulation in central nervous system involvement of tuberculosis, especially in tuberculous meningitis^24–26^. Besides, an elevated level of CSF protein is also known as an enhanced immunological reaction^27^. In the study, high level of CSF protein was identified as another risk factor for the poor outcome in children with TBM. As known, CSF examination of TBM patients showed low glucose, high protein, and pleocytosis^17,28^, and these CSF findings also served as criteria for the diagnosis of TBM. Previously, the association between CSF findings and outcome has been confirmed in adulthood TBM. For example, Hosoğlu et al. reported that high level of CSF protein was one of the five factors predicting the fatal outcome of TBM^29^. Similar finding was observed in our study. Our data suggested that high level of CSF protein is associated with poor outcome of childhood TBM. Besides this, several interesting findings about CSF protein were yielded recently. Such as faster normalization of CSF parameters (such as glucose, high protein, and pleocytosis) was associated with better outcome^28^; an increased level of CSF protein may cause more formation of basal exudates in patients with TBM, leading to cranial nerve involvement^30^.

The study has several limitations that should be taken into account. First, our study has a retrospective nature and a long inclusion period, during which diagnostic assays and treatment may have improved. Second, due to the observational and exploratory nature, the results may not be generalized to other settings. Third, due to the limited information collected from medical records and small sample size, our study did not address the relationship between GCS and outcome. Fourth, transfer to a higher-level hospital is defined as a poor outcome of childhood TBM. However, it does not mean that patient required transfer would experience death. Hence, our results should be interpreted with care. In a word, further analysis is needed to address the above mentioned questions and validate our findings in a large-scale prospective study.

## Conclusions

During the last decade, childhood TBM remains to have a high mortality rate in China. The study identified several risk factors of poor outcome, such as high level of CSF protein and coma. Childhood TBM remains a severe problem to the public health and more attention are required to initiate anti-TB therapy timely.

## Supplementary Information

Supplementary Information.
