# Supplementary material for: Risk factors for poor outcome in childhood tuberculous meningitis
Source: Sci Rep. 2021 Apr 21;11:8654. doi: 10.1038/s41598-021-87082-5 (PMC8060316; doi:10.1038/s41598-021-87082-5)
Supplement: Supplementary file 1 — Supplementary Information. [file 41598_2021_87082_MOESM1_ESM.docx]

**Risk factors for poor outcome in childhood tuberculous meningitis**

**Running title:** Factors influencing poor outcome in childhood TBM

Mao-Shui Wang^1,2^, Mei Zhao ^3^, Xin-Jie Liu^1^

Mao-Shui Wang, wangmaoshui@gmail.com

Mei Zhao, 1594350094@qq.com

Xin-Jie Liu, liuxinjie@sdu.edu.cn

^1^ Department of Pediatrics, Qilu Hospital, Cheeloo College of Medicine, Shandong University, Jinan, Shandong, China;

^2^ Department of Lab Medicine, Shandong Provincial Chest Hospital, Cheeloo College of Medicine, Shandong University, Jinan, Shandong, China;

^3^ Department of Pediatrics, Shandong Maternal and Child Health Hospital, Jinan, Shandong, China;

**Address correspondence to:**

Xin-Jie Liu, Department of Pediatrics, Qilu Hospital, Cheeloo College of Medicine, Shandong University, 107#, Wenhuaxi Road, Jinan 250012, China. Tel: +86 13964064194; Fax: +86 531 86927544; E-mail: [liuxinjie@sdu.edu.cn](mailto:liuxinjie@sdu.edu.cn).

| Supplementary Table 1. Univariate analysis of the demographic data associated with poor outcome in childhood tuberculous meningitis. | | | | | |
| --- | --- | --- | --- | --- | --- |
|  | | Total (n) | Poor group (n) | Good group (n) | P value |
| N | | 149 | 22 | 127 |  |
| Demographic characteristics | |  |  |  |  |
|  | Weight (Kg) | 24.2±16.0 | 22.2±17.6 | 24.5±15.8 | 0.557 |
|  | Rural area | 120 (80.5%) | 17 (77.3%) | 103 (81.1%) | 0.676 |
|  | T-SPOT.TB (+) | 26/38 | 3/5 | 23/33 | 0.665 |
|  | ESAT-6 (dots) | 63±94 | 125±141 | 53±84 | 0.132 |
|  | CFP-10 (dots) | 41±57 | 104±124 | 31±34 | 0.073 |
| Clinical Chemistry (serum) | |  |  |  |  |
|  | Total protein (g/L) | 68.8±7.8 | 69.2±11.3 | 68.8±7.2 | 0.447 |
|  | Albumin (g/L) | 40.3±4.9 | 39.5±6.1 | 40.4±4.7 | 0.487 |
|  | Blood urea nitrogen (mmol/L) | 4.47±3.12 | 5.69±5.39 | 4.28±2.58 | 0.344 |
|  | Creatinine (μmmol/L) | 38.2±16.0 | 35.8±15.0 | 38.5±16.1 | 0.260 |
|  | Glucose (mmol/L) | 4.99±0.94 | 5.24±1.02 | 4.94±0.93 | 0.500 |
|  | Lactate dehydrogenase (U/L) | 281±270 | 517±718 | 246±75 | 0.147 |
| Blood analysis | |  |  |  |  |
|  | White blood cell (10^9^/L) | 9.8±4.9 | 10.7±3.7 | 9.7±5.0 | 0.594 |
|  | Red blood cell (10^12^/L) | 4.34±0.43 | 4.42±0.48 | 4.33±0.42 | 0.422 |
|  | Hemoglobin (g/L) | 118±15 | 119±13 | 118±16 | 0.704 |
|  | Hematocrit | 33.2±8.6 | 33.7±9.3 | 33.1±8.5 | 0.775 |
|  | Mean corpuscular volume (fL) | 81.1±6.2 | 80.4±4.7 | 81.2±6.4 | 0.603 |
|  | Mean corpuscular haemoglobin (pg) | 27.2±2.7 | 27.0±1.6 | 27.2±2.8 | 0.784 |
|  | Mean corpuscular haemoglobin concentration (g/L) | 335±18 | 336±12 | 334±19 | 0.717 |
|  | Platelet (10^9^/L) | 354±123 | 370±138 | 351±121 | 0.562 |
|  | Neutrophil (10^9^/L) | 6.5±7.4 | 6.9±3.0 | 6.4±7.9 | 0.784 |
|  | Lymphocyte (10^9^/L) | 2.9±2.2 | 2.8±2.3 | 2.9±2.2 | 0.842 |
|  | Monocyte (10^9^/L) | 0.9±0.6 | 0.9±0.4 | 0.9±0.7 | 0.940 |
|  | Coefficient of variation of red cell distribution width (%) | 14.6±2.6 | 14.1±1.4 | 14.7±2.7 | 0.391 |
|  | Erythrocyte sedimentation rate (mm/h) | 25.3±23.4 | 28.1±19.7 | 24.9±23.9 | 0.885 |
| Flow cytometry | |  |  |  |  |
|  | CD19+ (%) | 24.9±11.3 | 36.1±22.4 | 23.4±8.2 | 0.065 |
|  | CD3+ (%) | 62.1±10.6 | 56.4±13.3 | 62.9±10.0 | 0.107 |
|  | CD3+CD4+ (%) | 32.0±8.4 | 28.7±7.7 | 32.5±8.5 | 0.230 |
|  | CD3+CD8+ (%) | 25.7±9.2 | 24.2±16.3 | 26.0±7.9 | 0.606 |
|  | CD3-CD16+CD56+ (%) | 9.7±5.9 | 9.8±4.1 | 9.7±6.2 | 0.986 |
|  | CD4+/CD8+ | 1.6±1.4 | 2.4±2.9 | 1.5±1.0 | 0.129 |
| TB, tuberculosis; OR, odds ratio; CI, confidence interval. | | | | | |
